# An Efficient Bifunctional Electrocatalyst of Phosphorous Carbon Co-doped MOFs

**DOI:** 10.1186/s11671-020-03394-x

**Published:** 2020-08-24

**Authors:** Li Du, Mengyuan Lv, Dandan Liu, Huiyu Song

**Affiliations:** The Key Laboratory of Fuel Cell Technology of Guangdong Province, School of Chemistry and Chemical Engineering, South China University of Technology, Guangzhou, 510641 China

**Keywords:** Bifunctional catalyst, ORR, OER, MOFs, C/P co-doped

## Abstract

It is eager to develop high-performance and cheap bifunctional electrochemical catalysts for both of the oxygen reduction reaction (ORR) or oxygen evolution reaction (OER) for the energy crisis and environmental problems. Herein, we report a series of ZIF-derived Co-P-C co-doped polyhedral materials with a well-defined morphology. The optimized catalyst Co/P/MOFs-CNTs-700 exhibited favorable electrochemical activities with the lowest overpotential of 420 mV to achieve the current density of 10 mA cm^−2^ for OER and the half potential of 0.8 V for ORR in 0.1 M NaOH. The performance can be well improved by doping phosphorous resource which greatly changed its morphology. Meanwhile, the doped carbon resources also improve the conductivity, which makes it a promising bifunctional electrochemical catalyst and can be comparable with the commercial electrocatalysts.

## Introduction

In recent years, the rapidly growing demand for energy sustainable development has drawn great interest among researchers in the field of electrochemical energy conversion and energy storage technologies [1–3]. In order to meet the demand for energy conversion and distribution, investigating an alternative non-precious metal electrode material with well-designed structure, controlled chemical, and excellent electrochemical performance will be a constant pursuit [4–7]. The oxygen evolution reaction (OER) and the oxygen reduction reaction (ORR) are the important reactions which act as significant roles in the application in solar cells, electrolysis cells, rechargeable metal-air cells, fuel cells, and so on [8–11]. Nevertheless, the dull kinetic of OER and ORR severely limited the large-scale utilization of the energy conversion efficiency [8, 12–15].

Therefore, great efforts have been put to explore an efficient and stable electrochemical catalyst to improve the severe oxygen reaction in the past decades. It is known that the noble metal catalysts are the benchmark of the oxygen reaction. However, these prospective materials suffered from scarcity, high cost, and low stability. For example, iridium dioxide and ruthenium dioxide, the most promising OER catalysts, exhibit excellent OER electrocatalytic activities both in acidic and alkaline conditions at low overpotential but still lacking long-term stability. The commercial platinum carbon catalyst as a kind of cathode electrocatalyst with the brilliant electrochemical activity of ORR is still cross-affected by the electrolyte, easy to be poisoned and lacks certain durability. Consequently, due to above shortcomings of the precious catalysts, more and more researchers have devoted to the design of electrochemical catalysts based on the abundant elements on the earth for the sustainable development. Interestingly, metal organic framework materials (MOFs) have attracted tremendous interests due to their low cost, abundant sources, and the ability to serve as templates for the synthesis of carbon-based nanoporous materials. The crystalline porous material MOFs are usually easy to design forming by self-assembly of metal ions and organic groups [16, 17]. The carbon-metal complexes derived from them can possess different morphologies, exhibiting extremely high surface areas and hierarchical pore structures which contribute greatly to the electrochemical activities of ORR and OER [18, 19]. Nonetheless, the graphitization degree of these materials is relatively low, thus reducing the conductivity of the materials.

Meanwhile, carbon nanomaterials due to their high conductivity and controllable morphology are extremely attractive and have been applied in many electrochemical devices, such as polymer fuel cell [2, 20]. Moreover, it has been proved that carbon nanomaterials doped with heterogenous elements can greatly enhance the catalytic activity and the surface chemistry area [4, 13, 20–26]. The hetero-doped carbon materials also have synergistic effect in direct catalysis of ORR [27, 28]. Therefore, in order to enhance the conductivity and catalytic activity of the materials, it is reasonable to synthesize efficient heterogenous atom-doped materials from inexpensive source-rich material MOFs, which can be well applied in fuel cells, metal-air batteries, and so on.

Therefore, we report an efficient bifunctional electrochemical catalyst of the metal organic framework with phosphorus and carbon co-doped by an in situ doping method. We have found that doping heterogenous atom can change its morphology and improve the conductivity proved by SEM and XPS, which made it process a favorably low overpotential of 420 mV to achieve the current density of 10 mA cm^−2^ for OER and the half potential of 0.8 V for ORR in 0.1 M NaOH. This promising bifunctional electrochemical catalyst can be comparable with the commercial electrocatalysts.

## Methods

### Synthesis of Co-MOF Carbon Nanomaterials

In order to synthesize the Co-MOF carbon nanomaterials, a typical and simple method was carried out as follows. Firstly, 1.28 g 2-methylimidazole was ultrasonically dispersed in 20 mL methanol to form solution A. 1.0 g cobalt(II) acetylacetonate was ultrasonically dispersed in 60 mL methanol to form solution B. The solution A was slowly added into solution B with continually ultrasonic for 5 min, followed by vigorous stirring for another 10 min at room temperature. Then, the mixture was sealed in the polytrafluoroethylene reactor, which was transferred into the air-dry oven and heated from room temperature to 160 °C and maintained at 160 °C for 24 h, followed by naturally cooling down to room temperature. The obtained purple solid powder was centrifuged and washed with methanol for several times and dried at 70 °C overnight. The prepared nanocrystals were pyrolyzed under an argon atmosphere in a flow-through quartz tube placed in the center of a tube furnace as follows. Firstly, the production was heated from room temperature to 350 °C at a rate of 5 °C/min and maintained at 350 °C for 1 h. Then, we increased it to the desired temperature (500, 600, 700, 800, and 900 °C) for 2 h with the same heating rate to obtain Co-MOFs-x, where “x” represents the carbonization temperature.

### Synthesis of Co/P-MOF Carbon Nanomaterials

In order to figure out the impact of doping P on the electrochemical activities, different phosphine sources were adopted during the synthesis. 1.28 g 2-methylimidazole was ultrasonically dispersed in 20 mL methanol to form solution A. 1.0 g cobalt(II) acetylacetonate and 0.25 g phosphorus source were ultrasonically dispersed in 60 mL methanol to form solution B. The phosphorus sources were sodium hypophosphite, triphenylphosphine, and *O*-trimethylphenyl phosphine. The following steps were the same as the above; we only changed the most suitable carbonization temperature as 700 °C. Finally, we obtained the productions named as Co/P0-MOFs, CoP1-MOFs, and Co/P2-MOFs, where P0, P1, and P2 represent sodium hypophosphite, triphenylphosphine, and *O*-trimethylphenyl phosphine, respectively.

We chose the triphenylphosphine as the phosphorus source and changed the mass of phosphorus source in step 1 to 0.5, 0.75, and 1.0 g, respectively. And the other experimental steps were unchanged. The final product was named Co/P/MOFs-700-0.25, Co/P/MOFs-700-0.5, Co/P/MOFs-700-0.75, and Co/P/MOFs-700-1.0, respectively.

### Synthesis of Co-MOFs-C Carbon Nanomaterials

In order to improve the conductivity of the material, additional carbon sources were added. 1.28 g 2-methylimidazole was ultrasonically dispersed in 20 mL methanol to form solution A. 1.0 g cobalt(II) acetylacetonate and 0.125 g carbon sources were ultrasonically dispersed in 60 mL methanol to form solution B. The carbon sources were carbon nanotubes (CNTs), acetylene black (CB), and A-OMCS that were prepared in our formal article [25] which were acid treated. The following steps were the same as the step 2.2 (1). Finally, we obtained the productions named as Co/MOFs-CNTs-700, Co/MOFs-CB-700, and Co/MOFs-A-OMCS-700, respectively.

### Synthesis of Co/P-MOFs-CNTs-700 Carbon Nanomaterials

In order to improve the conductivity and electrocatalytic performance, carbon sources and carbon materials were used synchronously at one time. 1.28 g 2-methylimidazole was ultrasonically dispersed in 20 mL methanol to form solution A. 1.0 g cobalt(II) acetylacetonate, 0.25 g triphenylphosphine, and 0.125 g CNTs with acid treated were ultrasonically dispersed in 60 mL methanol to form solution B. The following steps were the same as the above. Finally, we obtained the productions named as Co/P/MOFs-CNTs-700.

### Characterization of the Synthesized Carbon Nanomaterials

X-ray diffraction (XRD) was carried out on TD-3500 (Tongda, China) diffractometer. X-ray photoelectron spectroscopy (XPS) was carried out using a photoelectron spectrometer K-Alpha+ (Thermo Fisher Scientific). Scanning electron microscopy (SEM) images were obtained with an SU8220 scanning electron microscope (Hitachi, Japan). High-angle annular dark field (HAADF) imaging and energy-dispersive spectrometer (EDS) elemental mapping analysis were performed in the scanning transmission electron microscopy (STEM) mode on an aberration-corrected FEI Tecnai f20 field emission transmission; the electron microscope operated at 200 kV.

### Electrochemical Tests

All electrochemical activity data were collected on an electrochemical workstation (Ivium, Netherlands) at room temperature, coupled with a rotating disk electrode (RDE) system (Pine, USA) in a standard three-electrode system. The three-electrode system consisted of a Pt-wire counter electrode, a Hg/HgO (0.1 M NaOH solution) reference electrode for the alkaline medium, and a glassy-carbon-based working electrode (GC, 0.196 cm^2^). The catalyst-loaded electrodes were obtained as follows. Firstly, a catalyst ink was prepared by ultrasonicating a mixture of 1 mL of 0.25 wt % Nafion ethanol solution and 5 mg of the corresponding catalyst for 30 min. Then, 20 μL of catalyst ink was spread on a glassy-carbon-based working electrode in the RDE tests. Finally, the working electrode was dried under an infrared lamp for 1–2 min. The catalyst loading was approximately 0.5 mg cm^−2^. A 0.1 M NaOH solution was employed as the electrolyte and was purged with high-purity N_2_ or O_2_ gas for about 30 min before testing. Linear sweep voltammetry (LSV) tests were performed at a rotation rate of 1600 rpm and a potential scan rate of 10 mV s^−1^. Stability test was performed on Autolab Electrochemical Instrumentation (Metrohm) workstation in a standard three-electrode system, in which OER was by chronopotentiometry test conducted under constant current density of 10 mA cm^−2^ in 0.1 M NaOH with a loading of 0.2 mg cm^−2^ and ORR was by chronoamperometric response test performed under constant potential of 0.8 V under the same condition. All potentials are calibrated with respect to the reversible hydrogen electrode (RHE).

## Results and Discussion

**Fig. 1:**
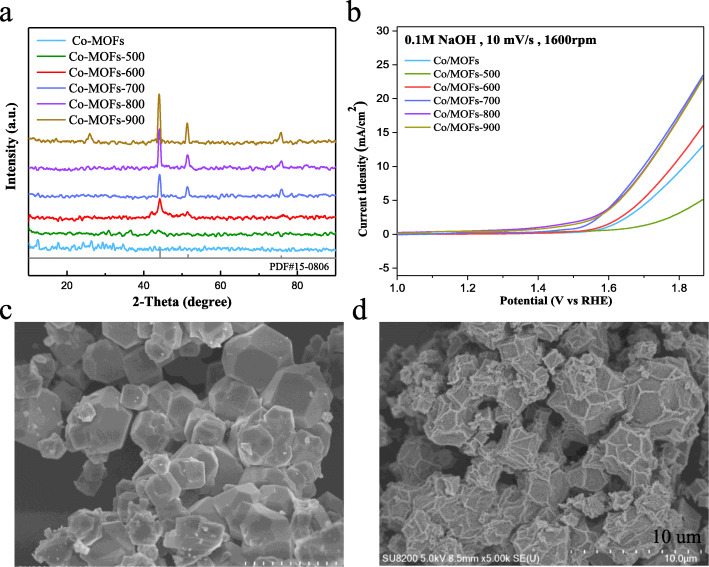
**a** XRD patterns of Co/MOF samples before carbonized and carbonized at different temperatures. **b** LSV curves for the OER of Co/MOFs, Co/MOFs-500, Co/MOFs-600, Co/MOFs-700, Co/MOFs-800, and Co/MOFs-900. **c**, **d** SEM images of Co/MOFs-700 sample before and after carbonized

As shown in Fig. 1a, X-ray diffraction (XRD) revealed that we have successfully synthesized polyhedrons of transition metal organic framework materials. When increasing the carbonization temperatures, the diffraction peaks at 44.216°, 51.522°, and 75.853° become more distinct, which matches well with the (111), (200), and (220) planes of the cubic cobalt (PDF#15-0806). As is known to all, the annealing temperature has a significant effect on the physicochemical and electrochemical performance of the samples [29, 30]. Thus, the obtained samples with a series of temperature gradient were conducted electrochemical measurements to examine the optimized temperature. Figure 1b shows the electrochemical activities of the materials treated at different temperatures. It is obvious to find that the as-prepared catalyst carbonized at 700 °C (Co/MOFs-700) exhibits the best OER performance. The overpotential is around 480 mV to achieve the current density of 10 mA cm^−2^ in 0.1 M NaOH.

Then, the Co/MOFs before carbonized and the best performed Co/MOFs-700 were picked to carry out the SEM measurements. As shown in Fig. 1c, d, the morphology of the obtained Co/MOFs-700 has changed greatly after carbonized at 700 °C. Many fold-like lines appear on its surface and are not smoother than the original materials without being carbonized. But it still processes the polyhedron morphology, with regular particle dispersion and no collapse sign.

As previous articles reported, doping the phosphor into transition metal organic framework polyhedron can increase the stability of the sample in acid or alkaline solution and can also effectively improve the electrochemical catalytic activity by breaking the electroneutrality and facilitating the O_2_ adsorption [31–33]. Therefore, phosphorus-doped samples are synthesized with in situ doping method and investigated the electrochemical performance. The obtained products were named as Co/P0/MOFs-700, Co/P1/MOFs-700, and Co/P2/MOFs-700, while the P0, P1, and P2 represent the phosphorus sources of sodium hypophosphite, triphenylphosphine, and *O*-trimethylphenyl phosphine, respectively.

**Fig. 2:**
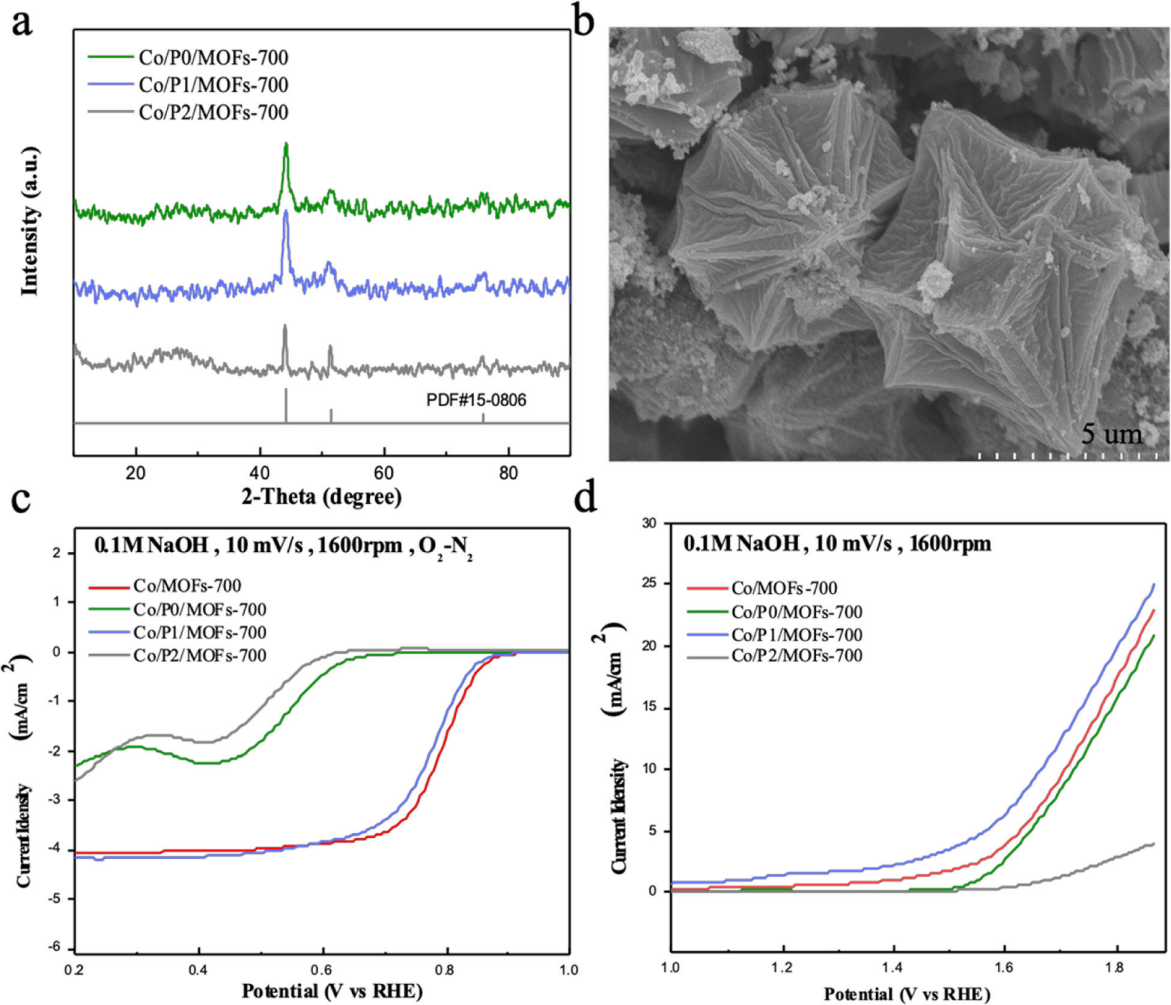
**a** XRD pattern of Co/P0/MOFs-700, Co/P1/MOFs-700, and Co/P2/MOFs-700. **b** SEM images of Co/P1/MOFs-700. **c** ORR polarization curves of the phosphorus-doped samples. **d** OER polarization curves of phosphorus-doped samples

According to Fig. 2a, the diffraction peaks of the phosphorus-doped samples still possess the pattern of cubic cobalt (PDF#15-0806), indicating that doping slight amount of phosphor would not change the structure of the MOFs. Then, the electrochemical measurements were conducted to explore the influence of different phosphorus sources on electrochemical catalytic activities. As shown in Fig. 2c, the open potential (0.87 V) and half wave potential (0.78 V) both show that the Co/P1/MOFs-700 possesses the best ORR activity. However, it is slightly weaker than that of the original product Co/MOFs-700 carbonized under the same temperature. Figure 2d represents the OER performance of different products doped with phosphorus. When the limited current density is 10 mA cm^−2^, only Co/P1/MOFs-700 owns the lowest overpotential of 430 mV, demonstrating that incorporation of phosphorus into the samples can increase the OER activity, which is coincidence with the reported article that incorporation of phosphorus would adjust the electrical conductivity and meanwhile facilitate the rapid electrons transfer [34]. Moreover, Fig. 2b shows a comparison between the sample with triphenylphosphine as phosphorus source and the original sample (Fig. 1c) without incorporation of elements. It can be revealed that the incorporation of phosphorus greatly affected the morphology of the material compared with Co/MOFs-700. Therefore, doping phosphor can not only enhance the electrochemical activity but also changed the morphology of the sample.

**Fig. 3:**
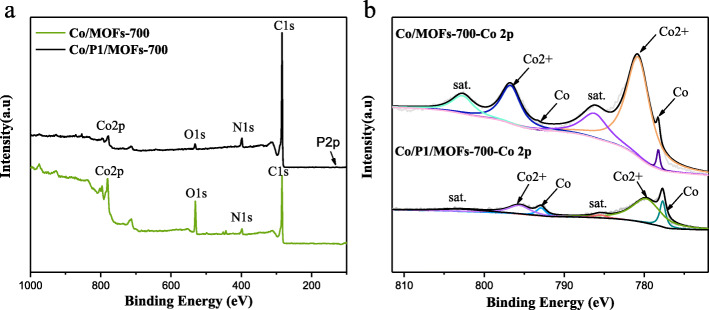
**a** XPS spectra survey of Co/MOFs-700 and Co/P1/MOFs-700. **b** Co 2p spectra of Co/MOFs-700 and Co/P1/MOFs-700

Subsequently, in order to further figure out the reason why doping phosphorus can enhance the electrochemical activity, XPS analysis was carried out to probe the composition and chemical state of the Co/MOFs-700 and Co/P1/MOFs-700 samples. According to Fig. 3a, XPS spectra survey of Co/MOFs-700 and Co/P1/MOFs-700 both shows the presence of Co 2p, O 1s, N 1s, and C 1s. It is noted that the peak of P 2p appears in the XPS spectra survey in Co/P1/MOFs-700 but shows a rather weak signal compared with strong peaks of C 1s. Moreover, Fig. 3b shows the Co 2p spectra of Co/MOFs-700 and Co/P1/MOFs-700. It was found that the Co 2p 3/2 can be fitted into two peaks. The peaks located at 778.2° and 780.7° can be ascribed to the Co (0) and Co (2^+^), while Co 2p 1/2 can also be displayed into two peaks positioned at 793.3° and 796.7°, which can be ascribed to the Co (0) and Co (2^+^). The satellite peaks were positioned at 786.2° and 802.7° [35–37]. When compared with phosphorus-doped sample Co/P1/MOFs-700, we can find that the Co (0) were greatly increased while Co (2^+^) decreased, indicating that doping phosphorus source during the synthesis process can increase the content of Co (0) in the obtained samples. As is known to us all, Co (0) can greatly enhance the conductivity, thus improving the electrochemical performances which is also in accordance with the previous report [38].

**Fig. 4:**
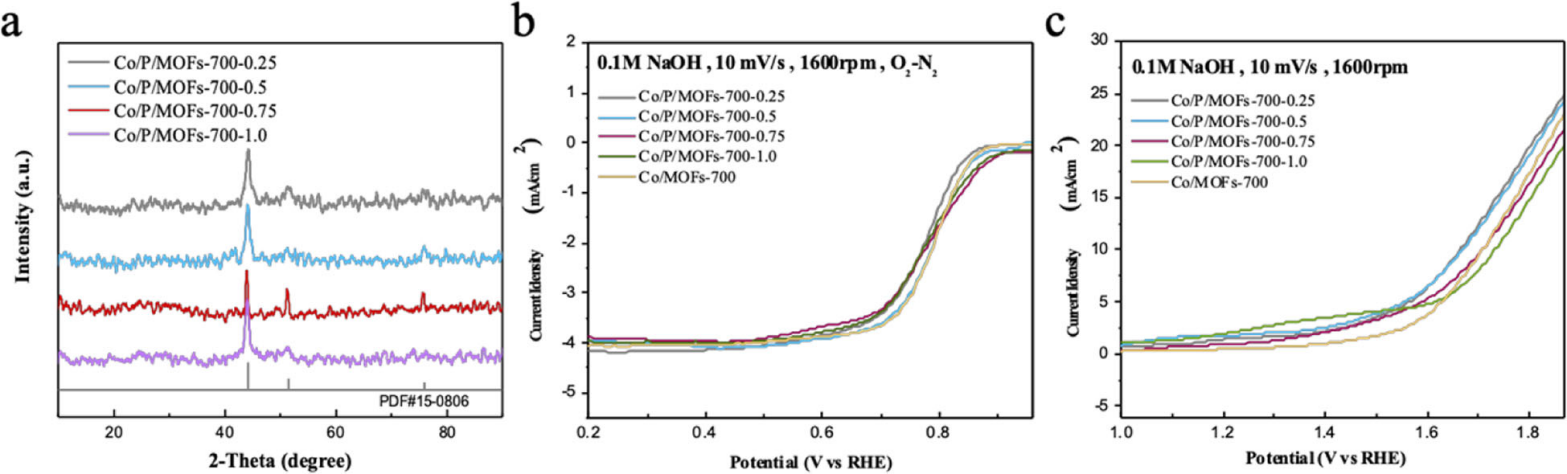
**a** XRD patterns of doping different contents of phosphorus source into Co-MOFs. **b**, **c** ORR and OER polarization curves of Co/MOFs-700, Co/P/MOFs-700-0.25, Co/P/MOFs-700-0.5, Co/P/MOFs-700-0.75, and Co/P/MOFs-700-1.0, respectively

Afterwards, we continued to investigate influence on the quality of the doped phosphorus source. The obtained products with a different molar ratio of P were named as Co/P/MOFs-700-x (*x* = 0.25, 0.5, 0.75, 1.0), while P represents triphenylphosphine and x represents the quality of phosphorus source. Figure 4a shows that when increasing the content of phosphorus sources, the XRD pattern shows that the main diffraction peaks in these samples are still cobalt (PDF#15-0806). As Fig. 4b shows, Co/P/MOFs-700-0.5 possesses the best ORR activity whose half wave potential was around 0.8 V among these phosphorus-doped products, but the ORR activity of Co/P/MOFs-700-0.5 is not increased significantly compared with the original sample Co/MOFs-700. It can be seen from Fig. 4c that the OER activity of the samples increased significantly with the addition of triphenylphosphine compounds and decreased with the increase mass of phosphorus source. When the limited current density is 10 mA cm^−2^, Co/P/MOFs-700-0.25 and Co/P/MOFs-700-0.5 both own the minimum overpotential of 450 mV, indicating that only proper amount of phosphorus sources can improve OER activity while the amount of 0.25 and 0.5 exhibits best. However, when compared with commercial platinum carbon (half wave potential 0.81 V, limiting current density 5.43 mA cm^−2^) and excellent OER electrocatalyst iridium oxide (1.61 V @ 10 mA cm^−2^), Co/P/MOFs-700-0.5 still remains significant difference among the limited current density in ORR performance. As the article reported, when the conductivity of the material is small, so is the limited current density [39].

**Fig. 5:**
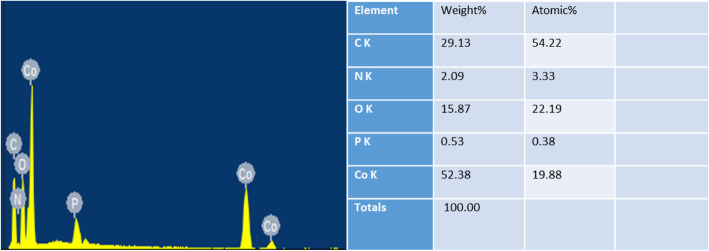
EDS of Co/P/MOFs-700-0.5

In order to enhance the conductivity, we firstly measured the current carbon content of the synthesized Co/P/MOFs-700-0.5 analyzed by EDS images. According to Fig. 5, it is obvious that the quality of cobalt accounts for the most which takes almost 52.38%, while the quality of carbon is relatively less of 29.13%.

**Fig. 6:**
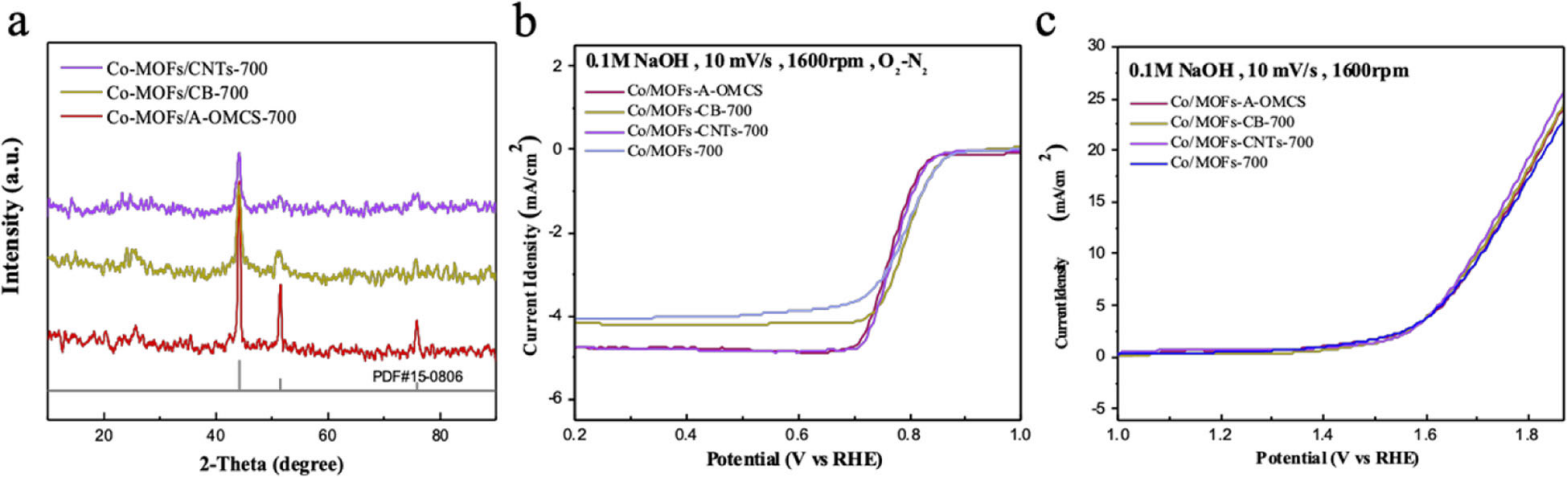
**a** XRD patterns of doping different contents of carbon source into Co-MOFs. **b**, **c** ORR and OER polarization curves of Co/MOFs-700, Co/MOFs-CNTs-700, Co/MOFs-A-OMCS-700, and Co/MOFs-CB-700, respectively

Therefore, in order to improve the conductivity of the material, we further doped the samples with carbon without any phosphorus sources. The obtained products were named as Co/MOFs-CNTs-700, Co/MOFs-CB-700, and Co/MOFs-A-OMCS-700, respectively. Figure 6a shows that doping carbon will not affect the structure of the samples, which still keeps the same diffraction peaks of cobalt (PDF#15-0806). As shown in Fig. 6b, it can be seen that the limited current density of the products is greatly increased with the incorporation of carbon source in ORR, while Fig. 6c indicated that the incorporation of carbon source makes no sense to improve the OER properties of the catalysts.

**Fig. 7:**
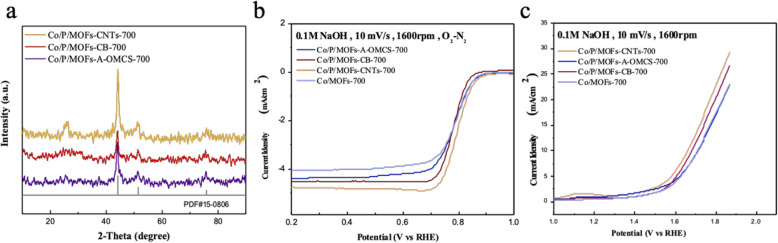
**a** XRD patterns of Co/P/MOFs-CNTs-700, Co/P/MOFs-A-OMCS-700, and Co/P/MOFs-CB-700. **b**, **c** ORR and OER LSV curves for the above samples, respectively

Combined with the previous experimental data and conclusions, we doped the original sample with both phosphorus and carbon elements by adding 0.5 g of triphenylphosphine and a suitable amount of different carbon sources (CNTs, CB, and A-OMCS) to the material for comparison. The obtained samples were named as Co/P/MOFs-CNTs-700, Co/P/MOFs-CB-700, and Co/P/MOFs-A-OMCS-700, respectively. According to Fig. 7a, there is no change of the XRD pattern, with all the samples matching well with cubic cobalt (PDF#15-0806). As shown in Fig. 7b, co-doping with phosphorus and carbon greatly increased the limited current density and ORR performance of the products. The sample of Co/P/MOFs-CNTs-700 exhibits the best ORR activity, in which the half wave potential and limiting current density are 0.8 V and 4.81 mA cm^−2^ and are 10 mV lower than that of commercial platinum carbon. Additionally, as can be seen clearly in Fig. 7c, the OER performance of the products has also been greatly improved. The sample of Co/P/MOFs-CNTs-700 exhibits the lowest overpotential voltage of 420 mV (Table 1). Compared with the voltage corresponding to dioxide iridium, Co/P/MOFs-CNTs-700 is only about 40 mV higher than dioxide iridium. Therefore, Co/P/MOFs-CNTs-700 exhibits to be a favorable bifunctional electrocatalyst.

**Table 1:** The comparisons of OER and ORR activities of as-prepared Co/P/MOFs-CNTs-700 with other superior hetero-doped MOFS or MOF-derived catalysts previously reported

Catalyst	OER, overpotential (mV@10 mA cm^−2^)	ORR, half potential (V)	Electrolyte concentration	Reference
**Co/P/MOFs-CNTs-700**	**420**	**0.8**	0.1 M NaOH	***This work***
Co_50_Zn_50_–C1100	440	0.82	0.1 M KOH	*Energy Environ. Sci.,* 2016, 9, 1661
Co_3_O_4_@C-MWCNTs	320	0.81	1 M KOH (OER) 0.1 M KOH(ORR)	*J. Mater. Chem. A 2015, 3, 17392*
Co/Co_3_O_4_–PC/CNTs	370	0.8	0.1 M KOH	*Angew. Chem., Int. Ed. 2016, 55, 4087*
Co_3_O_4_@Co/NCNT	380	0.86	0.1 M KOH	*Eur. J. 2017, 23, 18049*
CoNC@GF	430	0.87	0.1 M KOH	*Adv. Mater. 2018, 30, 1704898*
CoP@mNSP-C	410	0.9	0.1 M KOH	*Small. 2017, 13, 1702068*
Co/Co_x_S_y_@S,N-codoped porous carbon	370	0.76	0.1 M KOH	*ACS Appl. Mater. Interfaces 2017, 9, 39, 34269-34278*
Co@NSC	300	0.82	1 M KOH (OER) 0.1 M KOH(ORR)	*ACS Sustainable Chem. Eng. 2019, 7, 8, 7743–7749*
C-MOF-C2-900	350	0.82	0.1 M KOH	*Adv. Mater. 2018, 30, 1705431*
Co_3_O_4_/HNCP-40	333	0.83	0.1 M KOH	*ACS Catal. 2018, 8, 9, 7879–7888*

Listed is the brief summary for the state of the art of hetero-doped MOFS for OER and ORR

**Fig. 8:**
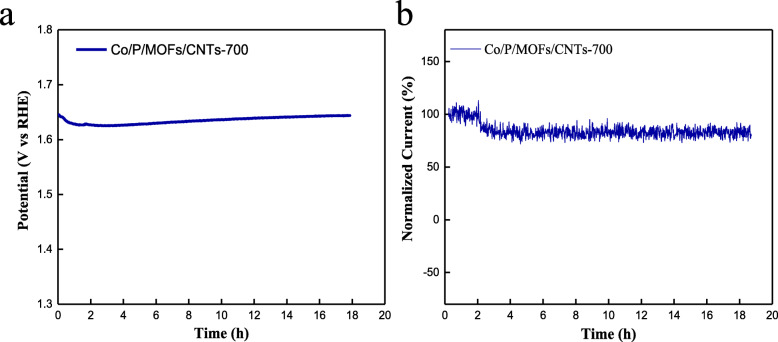
**a** Chronopotentiometry measurement for Co/P/MOFs-CNTs-700. **b** Chronoamperometric response of Co/P/MOFs-CNTs-700

Meanwhile, to access the stability of the best performed Co/P/MOFs-CNTs-700, chronopotentiometry and chronoamperometric response tests were carried out. As can be seen in Fig. 8a, b, the overpotential only increased 1.5 mV and the ORR performance is reduced by 79.5% after 18 h continuous tests, proving that both OER and ORR activities of Co/P/MOFs-CNTs-700 are rather stable in 0.1 M NaOH.

**Fig. 9:**
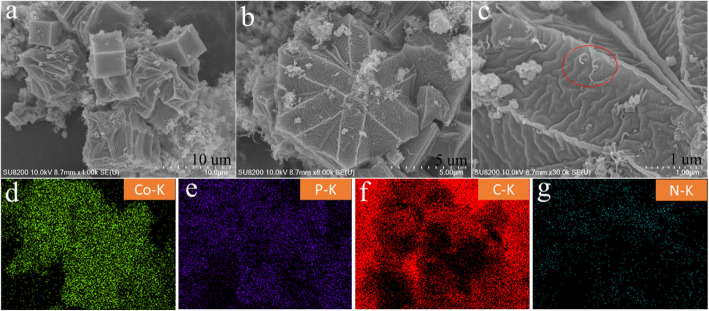
SEM images of Co/P/MOFs-CNTs-700 (**a**–**c**) and the corresponding elemental mapping of Co (**d**), P (**e**), C (**f**), and N (**g**), respectively

Scanning electron microscopy, EDS, and mapping on the sample of Co/P/MOFs-CNTs-700 have also been carried out. As can be seen from Fig. 9a–c, Co/P/MOFs-CNTs-700 retained the polyhedron morphology with many fold-like lines on the surface. Besides, the incorporation of carbon nanotubes is embedded into the skeleton of the product, which may increase the specific surface area of the product and provides more adsorption sites for electrochemical reaction. Figure 9 d–g are the mapping analysis of the sample. It can be seen that the carbon and phosphorus sources are uniformly dispersed in the skeleton of the sample and become whole.

**Fig. 10:**
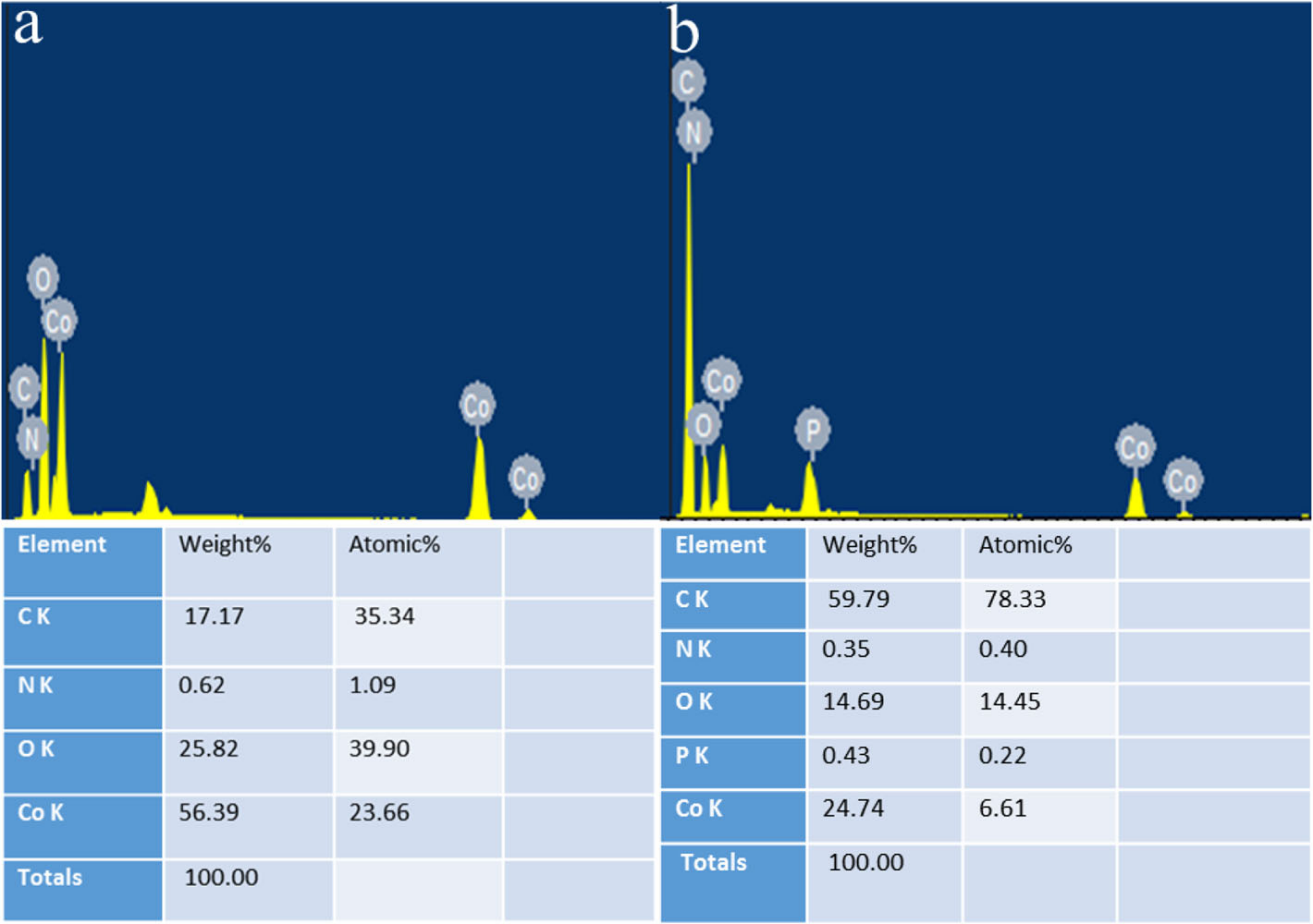
**a**, **b** EDS analysis of Co/MOFs-700 and Co/P/MOFs-CNTs-700, respectively

As EDS shows, the content of phosphorus and carbon of the material is increased compared with the original sample of Co/MOFs-700 by in situ doping, thus leading to the increased ORR and OER activity (Fig. 10). It is laterally demonstrated that the incorporation of two kinds of phosphorus and carbon elements could be beneficial to increase the electrochemical activity of metal organic framework materials containing cobalt [40], because the electronegativity of P (2.19) is different from that of carbon atoms (C, 2.55). Co-doping would break the electroneutrality which can facilitate the O_2_ adsorption and improve the ORR activity [41]. Meanwhile, more active sites can arise due to the co-doping phosphorus and carbon by changing the asymmetric spin density of heteroatoms and effectively weakening the O-O bonding, thus leading to the enhanced ORR activity [42].

The outstanding electrochemical activities can be attributed to the following reasons. Firstly, doping heteroatoms would lead to the redistribution of the charge density on the catalyst surface, which is beneficial to adsorb oxygen and promote the ORR activities [43]. Secondly, co-doping different atoms into the MOFs would result in the synergistic effect which also contributes to the enhanced electrochemical performance [44]. Thirdly, it has been proved that the OER mechanism of Co-based catalyst is a dynamic surface self-reconstruction process. The Co atoms on the surface could form a self-assembled metal oxy(hydroxide) active layer of CoOOH which works as a real active site [45]. In addition to the composition, the unique hybrid structure combined with its high conductivity could provide large surface area for the fast charge transfer.

## Conclusion

In conclusion, an efficient and cost-effective polyhedron transition metal organic framework carbon nanomaterial (Co/P/MOFs-CNTs-700) co-doped with phosphorus and carbon sources has been successfully synthesized, which can serve as an efficient and cheap bifunctional electrochemical catalyst. The lowest overpotential of Co/P/MOFs-CNTs-700 is 420 mV to achieve the current density of 10 mA cm^−2^ for OER, and the half potential is 0.8 V for ORR in 0.1 M NaOH, which is very close to those of commercial electrochemical catalysts. It could be utilized as a promising electrochemical bifunctional electrocatalyst in the energy storage field and also provide a promising insight to design electrochemical bifunctional electrocatalyst.

## Data Availability

The data used to support the findings of this study are included within the article.

## Abbreviations

ORR: Oxygen reduction reaction
OER: Oxygen evolution reaction
SEM: Scanning electron microscopy
HAADF: High-angle annular dark field
EDS: Energy-dispersive spectrometer
STEM: Scanning transmission electron microscopy
XPS: X-ray photoelectron spectroscopy
XRD: X-ray diffraction
RDE: Rotating disk electrode
Co-MOFs-x: Cobalt-metal organic frameworks-x represents temperature
Co/P-MOFs: Cobalt/phosphorus-metal organic frameworks
Co/P/MOFs-700-0.25: Cobalt/phosphorus-metal organic frameworks-700 °C-the mass of phosphorus source is 0.25
Co-MOFs-C: Cobalt-metal organic frameworks-carbon
Co/P-MOFs-CNTs-700: Cobalt/phosphorus-metal organic frameworks-carbon nanotubes-700 °C
GC: Glassy carbon
LSV: Linear sweep voltammetry
RHE: Reversible hydrogen electrode
Pt/C: Platinum/carbon catalyst
